# Revisiting the Motivated Denial of Mind to Animals Used for Food: Replication Registered Report of Bastian et al. (2012)

**DOI:** 10.5334/irsp.932

**Published:** 2024-04-26

**Authors:** Tyler P. Jacobs, Meiying Wang, Stefan Leach, Ho Loong Siu, Mahika Khanna, Ka Wan Chan, Ho Ting Chau, Katy Y. Y. Tam, Gilad Feldman

**Affiliations:** 1 Department of Psychology, Swarthmore College, USA; 2 Department of Marketing, London Business School, UK; 3 Department of Psychology, Lancaster University, UK; 4 Department of Psychology, The University of Hong Kong, Hong Kong SAR; 5 Department of Psychology, King’s College London, UK

**Keywords:** cognitive dissonance, mind attribution, mind denial, morality, meat, animals, registered replication

## Abstract

Bastian et al. (2012) argued that the meat paradox—caring for animals yet eating them—creates a tension between people’s moral standards (caring for animals) and their behavior (eating them) that can be resolved via mechanisms of motivated moral disengagement. One disengagement mechanism that is thought to play a central role is the denial of food-animal minds and therefore their status as moral patients. This idea has garnered substantial interest and has framed much of the psychological approach to meat consumption. We subjected Studies 1 and 2 of Bastian et al. (2012) to high-powered direct replications and found support for the target article’s hypotheses, concluding a successful replication. Perceptions of animals’ minds were negatively related to their perceived edibility (original: *r* = –.42 [–.67, –.08]; replication: *r* = –.45 [–.69, –.12]), positively related to moral concern for them (original: *r* = .77 [.58, .88]); replication: *r* = .83 [.68, .91]) and positively related to negative affect related to eating them (original: *r =* .80 [.63, .90]; replication: *r* = .80 [.62, .90]). Learning that an animal will be used for food led people to deny its mental capabilities (original: *d* = 0.40 [0.15, 0.65]; replication: *d* = 0.30, 95% CI [0.24, 0.37]), with the affect slightly weaker than the original. Our findings support the idea that the meat paradox is resolved through people’s motivated denial of food animals’ minds. Materials, data, and code are available on the OSF: https://osf.io/h2pqu/. This Registered Report has been officially endorsed by Peer Community in Registered Reports: https://doi.org/10.24072/pci.rr.100545.

## PCIRR-Study Design Table

**Table:** 


QUESTION	HYPOTHESIS	SAMPLING PLAN	ANALYSIS PLAN	RATIONALE FOR DECIDING THE SENSITIVITY OF THE TEST FOR CONFIRMING OR DISCONFIRMING THE HYPOTHESIS	INTERPRETATION GIVEN DIFFERENT OUTCOMES	THEORY THAT COULD BE SHOWN WRONG BY THE OUTCOMES

How are perceived mental capabilities of animals related to their perceived edibility?	H1a: Greater perceived animal mental capabilities will be associated with lower perceived edibility.	The current study aims to recruit 1000 participants, well-powered enough to detect effects much weaker than the smallest effects in the target. See Power analysis section.	Pearson Correlation	We follow the statistical analysis of the original paper.	We examine the replicability of the findings of Bastian et al. (2012) Studies 1 and 2 based on the criteria used by LeBel et al. (2019).	The meat paradox is facilitated by the denial of food animals’ minds.

How are perceived mental capabilities of animals related to negative affect of eating them?	H1b: Greater perceived animal mental capabilities will be associated with greater negative affect regarding eating them.					

How are perceived mental capabilities of animals related to moral concern for animals?	H1c: Greater perceived animal mental capabilities will be associated with greater moral concern for animals.					
	
How does learning that an animal will be used for food affect perceptions of its mental capabilities?	H2: Learning that an animal will be used for food will lead to reduced perceptions of that animal’s mental capabilities.		Paired-Samples t-Test			


As a society, we care for animals yet eat them. Loughnan et al. (2010) coined this phenomenon the meat paradox and explained it in terms of motivated moral disengagement driven by an aversive tension between people’s moral standards (caring for animals) and their behavior (eating animals). One mechanism that is thought to play an important role in resolving this tension is the motivated denial of food animals’ minds and therefore their capacity to feel pain and be harmed (Bastian & Loughnan, 2017; Loughnan & Davies, 2020). By positing that people are motivated to deny the minds of the animals they eat, Bastian et al. (2012) present a psychological explanation of how we can care for animals and simultaneously eat them. This idea has garnered substantial interest and has framed much of the psychological approach to meat consumption (Bastian & Loughnan, 2017; Dhont & Hodson, 2020; Loughnan & Davies, 2020; Piazza, 2020; Rothgerber, 2014). It therefore seems timely and worthwhile to revisit Bastian et al.’s (2012) seminal studies on the motivated denial of food animals’ minds.

## The Meat Paradox

How can people care for animals yet eat them? This seems paradoxical, given the wide and deeply held beliefs against harm (Graham et al., 2009; K. Gray et al., 2012), human fondness for animals (Amiot & Bastian, 2015; Kellert & Wilson, 1993), and the necessity of harming them to produce meat. Bastian and Loughnan (2017) provide an answer by drawing on Cognitive Dissonance Theory (Festinger, 1957; Harmon-Jones et al., 2015). They posit that individuals recruit psychological mechanisms that effectively resolve the aversive conflict between their beliefs and behaviors, thus escaping the paradox. These mechanisms are evident when, for example, meat-eaters derogate those who do not eat meat (De Groeve & Rosenfeld, 2022; De Groeve et al., 2022; Minson & Monin, 2012) and justify meat-eating as acceptable by virtue of it being ‘nice’, ‘necessary’, ‘normal’, and ‘natural’ (Piazza et al., 2015).

One psychological mechanism that is thought to play a particularly important role in minimizing cognitive dissonance and thus contributing to the meat paradox is people’s tendency to deny food animals’ minds (Bastian & Loughnan, 2017; Loughnan & Davies, 2020). This is because mental capacities, including the capacity to suffer, are grounds for moral status (Bentham, 1843; H. M. Gray et al., 2007; Leach et al., 2021a; Singer, 1975; Sytsma & Machery, 2012). The conflict between harming animals and eating them therefore depends on the perceived quality of their minds. When animals are perceived to lack minds, eating them is less morally fraught because they are less capable of being harmed (Leach et al., 2021a; Sytsma & Machery, 2012). Given that mind perception is malleable (Epley et al., 2008; Marcu et al., 2007), the tension between caring for animals and eating them can be resolved by seeing them as possessing unsophisticated minds and lacking the ability to feel suffering.

## Revisiting Bastian et al. (2012)

Bastian et al. (2012) presented three tests of motived mind denial to food animals. We focus our attention on the first two. In an initial study, they asked 71 students about their perceptions of 32 animals and found that the degree to which animals were perceived to be edible was positively related to beliefs that they lacked minds. In a follow-up study, they prompted 66 students to consider two animals, one that was destined to be taken to an abattoir and slaughtered for meat and one that was destined to be moved to a paddock and spend its time eating grass. They found that the animal that was destined to be slaughtered for meat was perceived to possess a less sophisticated mind than the animal that was destined to be moved to a paddock. Based on these findings, the authors argue that: 1) those animals that are perceived to be edible are also likely to be perceived as lacking a mind, and 2) making an animal’s status as a source of food salient can lead people to perceive it as lacking a mind. Taken together, the studies suggest that how we perceive animal minds is directly related to their status as sources of food.

## Rationale for Replication

We chose to conduct a replication of Bastian et al. (2012) due to its strong academic impact and the absence of direct replications. At the time of the writing, the target article has been cited 610 times (as indexed by Google Scholar, January 2024), and its findings and perspective have framed much of the subsequent psychological research on meat consumption (Bastian & Loughnan, 2017; Bratanova et al., 2011; Buttlar & Walther, 2018; Camilleri et al., 2020; Dhont et al., 2021; Dowsett et al., 2018; Graça et al., 2016; Haslam & Loughnan, 2014; Kunst & Hohle, 2016; Leach et al., 2021a, 2021b, 2022; Loughnan & Davies, 2020; Loughnan et al., 2010; Piazza, 2020; Piazza & Loughnan, 2016; Piazza et al., 2015; Rothgerber, 2014). These metrics indicate strong academic impact, therefore raising the importance of revisiting, reproducing, and replicating its methods and findings.

To the best of our knowledge, there are no published direct replications of the original article. We were able to identify two conceptual replications of Study 1: Ruby and Heine (2012) and Possidónio et al. (2019). Ruby and Heine (2012) found that perceptions of animals’ intelligence were positively related to feelings of disgust at eating them, while Possidónio et al. (2019), on the other hand, found no support for the link between perceptions of animals’ capacity to think or feel and their perceived edibility. The mixed results of conceptual replications and the absence of direct replications suggest the need to revisit the original studies.

We aimed to revisit the phenomenon to examine the reproducibility and replicability of the findings. Following the recent and growing recognition of the importance of reproducibility and replicability in psychological science (e.g., Brandt et al., 2014; Open Science Collaboration, 2015; van‘t Veer & Giner-Sorolla, 2016; Zwaan et al., 2018), we embarked on a well-powered pre-registered close replication of Bastian et al. (2012).

## Bastian et al. (2012): Findings

Bastian et al. (2012) tested and found support for several hypotheses derived from their account of the meat paradox. We summarized these in Table 1. In Study 1, they found that animals’ perceived mind was negatively related to their edibility (*r*(30) = –.42, 95% CI [–.67, –.08]), positively related to feeling bad about eating the animal (*r*(30) = .77, 95% CI [.58, .88]), and positively related to how morally wrong it would be to eat the animal (*r*(30) = .80, 95% CI [.63, .90]). In Study 2, they found that meat eaters attributed less mind to an animal after being informed that it would be used for food compared to not using it for food, *t*(65) = 3.24, *d* = 0.40, 95% CI [0.15, 0.65].

**Table 1:** Bastian et al. (2012) Studies 1 and 2: Summary of hypotheses.


HYPOTHESIS	PREDICTION

1a	Mind attribution is negatively associated with perceived edibility of animals.

1b	Mind attribution is positively associated with negative affect regarding eating animals.

1c	Mind attribution is positively associated with moral concern for animals.

2	Being told that animals will be raised for meat consumption (compared to being told they will live as grazing animals) leads to denial of mind for those animals.


## Overview of the Replication

Bastian et al. (2012) conducted three experiments. Our replication focused on Studies 1 and 2, which were simpler in design and can be administered to our target sample. We combined the two studies into a singular data collection, displayed in random order with some slight adjustments. This design allowed us to both test the designs of the original studies and run further tests to compare the effects of the different studies with the potential for additional insights. We have successfully employed similar designs in previous replications in our team (e.g., Adelina & Feldman, 2021; Vonasch et al., 2023; Yeung & Feldman, 2022). Also, we added one manipulation check item per condition in Study 2 and two attention check items (Aust et al., 2013) at the end of the survey in order to encourage and measure attentive participant engagement.

## Pre-Registration and Open-Science

We provided all materials, data, and code at: https://osf.io/h2pqu/. This project received a Peer Community in Registered Reports Stage 1 in-principle acceptance (https://rr.peercommunityin.org/articles/rec?id=190; https://osf.io/cru4z/), after which we created a frozen pre-registration version of the entire Stage 1 packet at (https://osf.io/azb3g/) and proceeded to the data collection stage. It has then gone through peer review and has been officially endorsed by the Peer Community in Registered Reports (Chambers, 2024; https://doi.org/10.24072/pci.rr.100545). All measures, manipulations, and exclusions conducted for this investigation are reported, and data collection was completed before conducting the data analyses. This Registered Report was written based on the Registered Report template by Feldman (2023).

## Method

### Power Analysis

To ensure the current replication sample had sufficient power, we calculated effect sizes and confidence intervals (CI) based on the statistics reported in the target article with the help of a guide by Jané et al. (2024). To account for possible overestimation of effect sizes, we conducted a conservative power analysis using the ‘safeguard’ method (Perugini et al., 2014) in R (R Core Team, 2022) with the <pwr> package, which uses the lower bound of 60% CI of the original effect size. The required sample sizes for Studies 1 and 2 were determined by analyzing the smallest effect size from each study. More details on calculations and results are given in the Power Analysis section of the Supplementary Materials.

The results of the power analyses suggested that the sample size should be 112 in Study 1 to have a 95% probability of detecting the safeguard effect size: *r =* –.33. However, we modified the original’s design for each participant to only rate 8 out of the 32 animals (see Table 4). To account for this, we multiplied by four, resulting in a total sample size of 448. For Study 2, we estimated the power for the within-subject design based on the safeguard effect size Cohen’s *d =* 0.29. As a result, 157 participants are required in Study 2. The largest sample size required from the two studies is 448.

Eventually, we decided to aim for a much larger sample size of 1,000 in our data collection, addressing any possible loss of power that may have resulted from the deviations in our study design from the original studies. First, Study 1 resulted in a multi-level data structure where each participant rated multiple animals. The power analysis described above does not take the multi-level nature of the data into account. Second, this replication has combined Studies 1 and 2 from Bastian et al. (2012) into one single data collection, which has the potential to introduce carry-over effects since each participant is responding to two sets of dependent variables rather than one. To account for these uncertainties, deviations, and possible exclusions, we decided to collect a sample size of 1,000. A sensitivity analysis conducted using the <pwr*>* package in R indicated that a sample of 1,000 participants at 95% power would be able to detect minimum effect sizes of *r* = .11 and *d* = 0.11, which are much smaller than the safeguard effect sizes and correspond to weak effects in social psychology research (Jané et al., 2024) (see the Power Analysis section of the Supplementary Materials for more details).

### Participants

We collected data from 1,000 participants using Prolific, an online participant recruitment platform commonly used in social science research (Palan & Schitter, 2018). To ensure that our sample only included meat-eaters, we used Prolific’s ‘Diet’ filter to exclude vegans and vegetarians. To verify that participants were indeed not vegetarians or vegans, participants completed the following item in the funneling section at the end of the survey: *Please indicate: Do you eat meat?* with options ‘*Yes, I eat meat’* and ‘*No I do not eat meat*’. 16 participants reported that they did not eat meat and were excluded according to our preregistration. Additionally, to ensure data quality and generalizability, we included only participants with a 95% or greater approval rate and used a Prolific option of a gender-balanced sample. After preregistered exclusions, 959 participants were included in analyses (*M*age = 40.00, *SD* = 14.00; 484 females, 464 males, 10 others, and 1 rather not disclose). We provided a comparison of the target article sample and the replication samples in Table 2.

**Table 2:** Differences and similarities comparing the target article and the replication.


	BASTIAN ET AL. (2012)	REPLICATION

Sample size	Study 1: 71 (after exclusion 63); Study 2: 66	1000 (959 after exclusion)

Geographic origin	Australian	Prolific (US)

Gender	59 Females; 12 Males (before exclusion; not specified after exclusion)	284 females, 264 males, 10 others, and 1 rather not disclose

Median age (years)	N/A	37.00

Mean age (years)	19.13	40.00

Standard deviation of age (years)	N/A	14.00

Age range (years)	17–29	18–93

Medium (location)	Australian University	Computer (online)

Compensation	N/A	1.90 USD

Year	2010 (estimate)	2022


We first pretested the survey duration with 30 participants to make sure our time-run estimate was accurate and then adjusted the pay as needed. The data of the 30 participants was not analyzed separately from the rest of the sample other than to assess survey completion duration and needed pay adjustments. The final assignment pay was $1.90 USD.

### Design and Procedure

We summarized the overall design for Studies 1 and 2 in Table 3. Additional details, summaries, and all measures are provided in the Supplementary Materials and survey files on the OSF.

**Table 3:** Summary of study design and materials.


**Study 1 Replication**	**Animals** 8 out of the following 32 (within-subject): 20 mammals: Bull, Pig, Goat, Kangaroo, Rabbit, Deer, Horse, Wolf, Dolphin, Dog, Cat, Elephant, Lion, Monkey, Gorilla, Rat, Antelope, Squirrel, Mole, Sloth 3 birds: Sparrow, Chicken, Pigeon 2 fish: Fish, Shark 3 crustaceans: Prawn, Crab, Lobster 1 amphibian: Frog 1 reptile: Turtle 1 mollusk: Snail 1 insect: Housefly

	**DV1: Mental Capacities** The degree to which each animal possessed 10 mental capacities (1 = *Definitely does not possess*, 7 = *Definitely does possess*; α = .81–.91 for different animals) 10 mental capacities: hunger, fear, pleasure, pain, rage, self-control, morality, memory, emotion recognition, planning **DV2: Animal Edibility** “Would you choose to eat this animal” and “Would you eat this animal if asked to?” (1 = *Definitely would not*, 7 = *Definitely would*) **DV3: Negative affect** “How bad would you feel if you ate this animal?” (1 = *Not at all*, 7 = *Extremely*) **DV4: Moral concern** “How morally wrong would it be to eat this animal” (1 = *Not at all*, 7 = *Extremely*)

**Study 2 Replication**	**IV (Within-Subjects): Animal Use Condition** **Food condition:** Description that the animal will be taken to an abattoir and butchered as a meat product for human consumption. **Nonfood condition:** Description that the animal will be moved to other paddocks and will spend most of its time eating grass with other animals.

	**DV: Perceived Animal Mental Capabilities** “To what extent does this animal possess the following mental capacities?” for 15 mental capacities (pleasure, fear, rage, joy, happiness, desires, wishes, planning, goals, pride, pain, hunger, tasting, seeing, hearing)(1 = *Definitely does not possess*; 7 = *Definitely does possess*; α = .88–.91 for different animals)


**Table 4:** Summary of deviations between target article and current replication.


DEVIATIONS	TARGET ARTICLE (BASTIAN ET AL., 2012)	REPLICATION	REASON FOR CHANGES

Study 1: Number of animals to rate	32 animals.	Randomly select **8** out of 32 animals for each participant; we multiplied the required sample size by 4 in order to compensate for the modification.	Shorten survey, decrease participants’ cognitive load.

Study 1: List of Animals	32 animals including “cow” and “sheep”.	Replaced “cow” and “sheep” with “**Ox**” and “**Pig**”.	Avoid repetition of animals (Studies 1 and 2 were combined).

Study 1: Measure Wordings	In Study 1, animal edibility item: “Would you choose to **each** this animal?”.	“**each**” was changed to “**eat**”.	A typo was detected in the original article.

Study 2: 5 min unrelated filler task	There was a 5 min unrelated task between two pictures in Study 2.	We did not implement 5 min unrelated filler task.	There was no indication of what that task was, and it was not indicated as theoretically or empirically important. Online participants have limited cognitive capacity and patience for long surveys, so we removed the unrelated filler task as a tradeoff for data quality.

Study 2: Manipulation Wording	In Study 2, the original manuscript was inconsistent regarding whether one of the animals was a sheep or lamb.	We used the label that was used in the actual original materials (“lamb”).	Following a helpful reviewer comment, we note that future work could determine if different labelling (sheep vs. lamb) might alter the results.

Study 2: Manipulation Check	No manipulation checks were included.	We included a manipulation check for each level of the IV.	It is possible that participants could miss the manipulated caption, particularly online. Thus, manipulation checks measure if participants read the scenarios carefully.

Studies 1 and 2: Item Randomization	Unclear if items for mental capacities in both Studies 1 and 2 were randomized.	Items for the mental capacities in both Studies 1 and 2 were **randomized**.	Prevent bias introduced by order and/or survey fatigue.

Attention Check	No attention checks were included.	Two attention checks were included.	Measures whether participants carefully read survey items, which can be an issue with online research.

Exclusion	Vegetarians were excluded at the end of the Study 1 survey. Exploratory analyses using exclusion criteria.	Vegetarians were excluded from participation in the survey, rather than just post-hoc. See the Exclusion Criteria section of the Supplementary Materials.	The research aims at non-vegetarians and non-vegans. Additional verification questions were added to increase data quality. We pre-registered that if we failed to find support for the hypotheses, we would also have examined the results using our exclusion criteria.


First, participants answered a question indicating that they consented to completing studies with attention, comprehension, and manipulation checks. Then, participants began the main studies. Both Studies 1 and 2 in Bastian et al. (2012) were combined into a single survey, and the presentation order of Studies 1 and 2 was randomized and counterbalanced. The materials and procedure for each study are described below. At the end of the survey, participants were asked to answer some demographic questions. Summary tables and detailed experimental instructions for Studies 1 and 2 procedures are available in the Supplementary Materials (see Table S8).

### Study 1 Materials

Participants rated 8 animals randomly selected out of a list of 32 animals. The animals listed in the survey were the same as the ones in the original study, except for two. “Ox” and “Pig” replaced “Cow” and “Sheep” due to the repetition of animals in Study 2. The list of animals is provided in Table 3. Participants were asked to rate each animal’s mental capacities (10 items), edibility (2 items), negative affect about eating it (1 item), and how morally wrong it would be to eat it (1 item).

### Study 2 Materials

#### Pictures With Descriptions

Participants were presented with pictures of an animal surrounded by grass (the images can be found in Figure S1 of the Supplementary Materials). The animals were a cow and a lamb, which were randomly assigned to either the nonfood or food conditions. In other words, if a participant first saw a lamb depicted as a nonfood animal, then the cow would later be depicted as the food animal, and vice versa. Prior to each picture was a description of the animal, which was manipulated to describe the animal as the source of the meat product or not. In the nonfood condition that appears first, the description for the animal reads, “*This lamb[cow] will be moved to other paddocks, and will spend most of its time eating grass with other lambs[cows]*.” In the food condition, the description reads, “*This lamb[cow] will be taken to an abattoir, killed, butchered, and sent to supermarkets as meat products for humans*.” Below each picture, participants were asked to rate the perceived mental capacities of the animal (see Table 3).

#### Manipulation Checks

In order to ascertain whether participants carefully read the manipulation and to assess whether the manipulation was effective, we included manipulation checks in each condition that participants completed after rating the mental capabilities of each animal. This was not included in the original study, yet we felt it was important to measure if participants read and understood the manipulation because factual manipulation checks such as the ones used in this study can increase attentiveness without weakening the experimental effect (Kane & Barabas, 2019). Our manipulation checks consisted of the following question, *“To make sure that you’ve read and understood the scenario, in the described scenario, what was the fate of the animal?”* There were three possible answers: “*It was sent to other paddocks to eat grass with other animals”*, “*It was released to live in a forest*”, or “*It was butchered and treated as a meat product*”. Nine participants failed at least one manipulation check.

#### Attention Checks

Two attention checks were used to measure participant attentiveness for use as an exploratory exclusion criterion, particularly because participant attention is sometimes reduced during online studies (Aust et al., 2013). The first was a logical statement attention check (Abbey & Meloy, 2017) that has been used in past research (Jacobs & McConnell, 2022). The check consists of one question in which participants select which everyday activities they have performed in the last week from a list. One of the items is “*Used a computer, tablet, or mobile phone”*. Participants should select this item because using a computer, tablet, or mobile phone is required to complete the study (the complete measure can be found in the Supplementary Materials under Attention Check Questions). Failing to select this item could be a possible reason for exclusion in analyses. The second attention check is an honesty check (Abbey & Meloy, 2017), in which participants respond to the item “*How serious were you in filling out this questionnaire?*” on a 1 (*Not at all*) to 5 (*Very much*) scale. Low scores indicate that participants self-reported that they were not taking the study seriously. We pre-registered an exploratory analysis if we had failed to find support for the findings (see the Exclusion Criteria section of the Supplementary Materials for more details on exclusions). Aiming to examine any potential data issues, we would have examined the results with failed attention and comprehension checks excluded. Given the successful replication, we did not conduct these analyses.

### Deviations From the Original

Since this replication combined Study 1 and Study 2 of the original study together, research designs were modified. We summarized additional deviations between the original study and our replication in Table 4.

### Evaluation Criteria for Replication Findings

We aimed to compare the replication effects with the original effects in the target article using the criteria set by LeBel et al. (2019) (see section ‘Replication Evaluation’ in Supplementary Materials). We pre-registered our criteria for the conclusion of a successful replication. For Study 1, it is a successful replication if all three hypotheses (1a–1c) are supported, a mixed replication if only one or two of the hypotheses are supported, and a failed replication if none of the hypotheses are supported. Study 2 is a successful replication if Hypothesis 2 is supported.

### Replication Closeness Evaluation

We provided details on the classification of the replications using the LeBel et al. (2018) criteria in Table 5 (see section ‘Replication Closeness Evaluation’ in the Supplementary Materials for details on this criteria). We summarized the replication as a ‘very close’ replication.

### Data Analysis Strategy

#### Replication

We conducted statistical analyses in accordance with the tests reported in the original article: correlational tests for Study 1 and paired t-tests for Study 2. All analyses used two-sided significance tests.

We note that while analyzing the methods used in the target article, we noticed an error in Study 2, which reported an independent-samples *t*-test. However, the within-subjects research design and reported degrees-of-freedom both indicated that a paired-samples *t*-test was used. We then contacted the first author, who verified that reporting it as an independent-samples *t*-test was a typo and that the reported result was indeed from a paired-samples *t*-test.

#### Additional Analyses

To better explore some nuances of the combined studies and the animals used, we conducted several exploratory analyses (results for these analyses can be found in the Supplementary Materials). First, in the original article, the authors only examined the relationships between mind perception, edibility, negative affect, and moral concern at the animal level. As an exploratory analysis, we also examined these correlations at the participant level to see if the hypothesized patterns were found for participants’ ratings of animals more generally. Second, in Study 2, we used a 2 × 2 mixed factorial ANOVA with animal food status (food vs. nonfood) as the within-subjects factor, animal species (cow-first vs. lamb-first) as the between-subjects factor, and perceived animal mental capacities as the dependent variable in order to determine any effects of animal species. If the animal species order makes a meaningful difference or if there is an interaction, it would suggest that participants are judging cows and lambs differently and that perceptions of meat animals’ minds vary by species and should be tested separately in future research. Next, we examined Pearson correlations between the Study 1 and Study 2 measures to examine the degree to which the combined studies were associated., with a positive correlation indicating that participants were responding to the studies similarly.

Additionally, we used moderated multiple regression analyses to test if study order moderated the results of Study 1 and a mixed ANOVA to test if study order moderated the results of Study 2. We also reran the primary analyses, considering only those participants for whom the study was displayed first.

## Results

### Study 1

**Figure 1:**
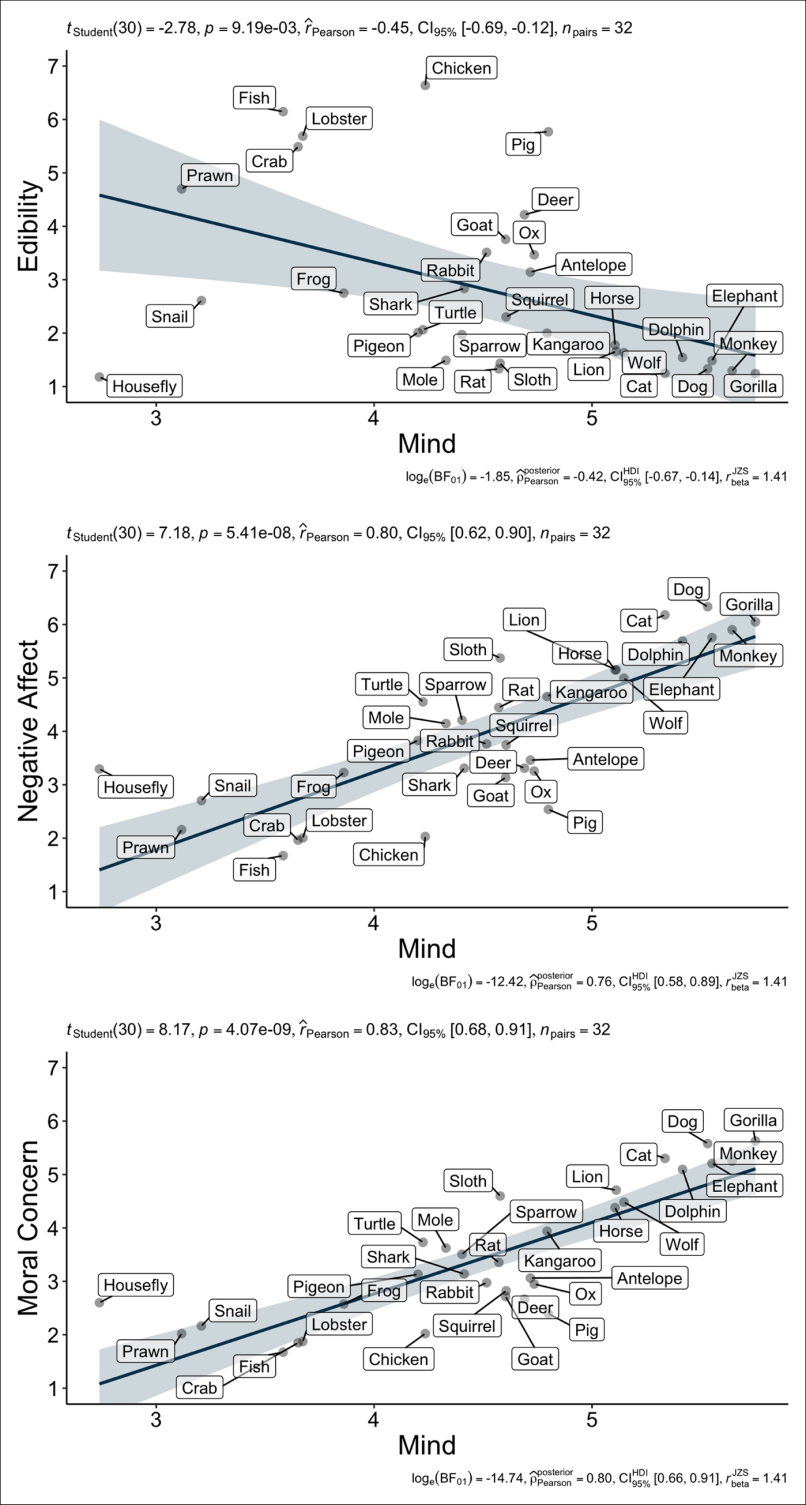
Study 1: Scatterplots of perceived mental capabilities’ associations with edibility, negative affect, and moral concern at the animal level.

As in Bastian et al. (2012), we aggregated responses across 959 participants and calculated animal-level means for the four dependent variables. Across the 32 animals, we obtained the mean mental capacity (*M* = 4.53, *SD* = 0.76), edibility (*M* = 2.80, *SD* = 1.66), negative affect (*M* = 4.00, *SD* = 1.38), and moral concern scores (*M* = 3.47, *SD* = 1.22). Scatterplots were created using the ggstatsplot package in R (Patil, 2021). Examining the associations between measures, we found support for all hypotheses. As seen in Figure 1, attributions of mind to animals were negatively associated with perceptions of edibility (H1a; *r*(30) = –.45, *p* = .009, 95% CI [–.69, –.12]). Attributions of mind to animals were positivity related to feeling bad about eating animals (H1b; *r*(30) = .80, *p* < .001, 95% CI [.62, .90]) and positivity related to moral concern for animals (H1c; *r*(30) = .83, *p* < .001, 95% CI [.68, .91]). Overall, the findings convincingly replicated Study 1 from Bastian et al. (2012).

### Study 2

**Figure 2:**
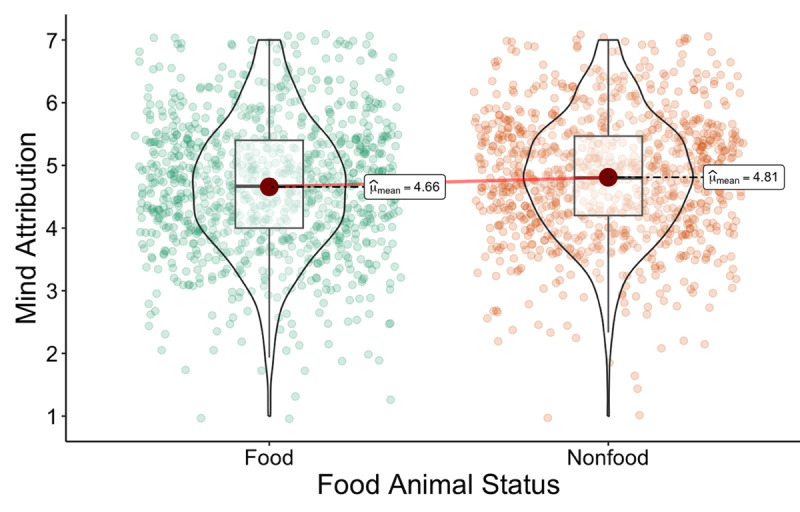
Study 2: Violin plot of the effect of animal food status on perceived animal mental capacities.

As seen in Figure 2, being informed that an animal would be used for food led to lesser perceptions of the animal’s mental capacities (*M =* 4.66*, SD =* 1.06) compared to being informed that an animal would not be used for food (*M =* 4.81*, SD =* 0.97), *t*(958) = 9.36, *p* < .001, *d* = 0.30, 95% CI [0.24, 0.37]. Using LeBel et al.’s (2019) criteria, the effect size was slightly weaker than in the original article (*d* = 0.40), yet the effect was detected and in the same direction. Thus, we concluded a successful replication of Study 2 from Bastian et al. (2012).

### Additional Analyses

We also examined the additional analyses aimed at further exploring the robustness and generalizability of the results. First, we conducted participant-level analyses for Study 1. Mean scores for mental capacities, edibility, negative affect, and moral concern were collapsed across animals for each participant. We then conducted Pearson correlations to assess the relationship between perceived mental capacities and perceived edibility, negative affect, and moral concern at the participant level. We summarized the results of these analyses in Table 6. Greater perceived mental capacity was associated with less perceived animal edibility. Greater perceived mental capacity was also associated with feeling worse about eating animals and a greater sense that it would be morally wrong to eat the animal. The effects were in the same direction as the animal-level analyses and supported the hypotheses, although they were smaller in size.

**Table 5:** Classification of the replication closeness, based on LeBel et al. (2018).


DESIGN FACET	REPLICATION	DETAILS OF DEVIATION

Effect/hypothesis	Same	–

IV construct	Same	–

DV construct	Same	

IV operationalization	Similar	In the Study 1 replication, each participant rated **8** animals randomly selected out of 32 instead of rating all 32 animals.

DV operationalization	Similar	We randomized the presentation order of the mental capacity items in both Studies 1 and 2.

IV stimuli	Similar	In Study 1 replication, animal items “**sheep**” and “**cow**” were changed to “**pig**” and “**ox**”, given that the same animals were rated in Study 2.

DV stimuli	Similar	In Study 1 replication, one of the items on edibility, “Would you choose to **each** this animal?”, was corrected to “Would you choose to **eat** this animal?”

Procedural details	Different	1) In the original study, participants in Studies 1 and 2 were separately recruited. Whereas in our replication, the same participants participated in both Studies. 2) The unrelated task between the cow/lamb ratings in Study 2 was eliminated. 3) Vegetarians and vegans were excluded from participation in the survey instead of at the end of the survey. 4) Manipulation and attention checks were added to the replication.

Physical settings	Different	In the original study, it was conducted on an Australian university campus. Whereas in our replication, the study was conducted on Qualtrics, completed by online Prolific participants.

Contextual variables	Different	

Replication classification	Very close replication	Based on the above analysis, we summarized our replications as a “very close” replication of the original studies.


*Note*. *N* = 959.

**Table 6:** Study 1: Summary of means, standard deviations, and correlations with animals’ perceived mental capacities at the participant level.


VARIABLE	*MEAN*	*SD*	*r*	*P*	*95% CI Upper*	*95% CI Lower*

Mental Capacities	4.53	1.00	–	–	–	–

Animal Edibility	2.79	1.18	–.06	.046	–.001	–.13

Negative Affect	4.00	1.55	.25	<.001	.31	.19

Moral Concern	3.47	1.61	.24	<.001	.29	.17


*Note*. *N* = 959.

We also conducted analyses exploring whether the order of animals presented in Study 2 moderated the effects on mind attribution and if study order moderated the effects of either study. We did not find any indication of order impacting any of the results or that whether the cow or lamb was presented first in Study 2 moderated the effects on mind attribution. We provided more details about the order analyses in the Supplementary Materials.

## Discussion

We conducted a replication of the Registered Report of Studies 1 and 2 of Bastian et al. (2012), examining whether viewing animals as food objects depresses mind attribution. We found support for all of the target article’s hypotheses (see Table 7). In our replication of Study 1, we found that attributions of mind were negatively related to animals’ edibility (H1a), positively to negative affect towards eating them (H1b), and positively to moral concern for them (H1c). In our replication of Study 2, we found that learning that an animal would be used for food led participants to attribute less mind to the animal (H2). For Study 1, the effect sizes were remarkably similar to the original. Additional analyses conducted at the participant-level found results that were in the same direction as the animal-level analyses but weaker in size. The effect size for Hypothesis 2 was slightly weaker compared to the target article’s, yet the effect was detected and in the same direction. Additional analyses found no indication that the order of the studies in the unified design impacted the results. Overall, we conclude a successful replication of Bastian et al. (2012).

**Table 7:** Bastian et al. (2012) Studies 1 and 2: Summary of replication based on LeBel et al. (2019) criteria.


H	HYPOTHESIS DESCRIPTION	METHOD	BASTIAN ET AL. (2012)’S EFFECT SIZE	REPLICATION EFFECT SIZE	REPLICATION EVALUATION

1a	Mind attribution is negatively associated with perceived edibility of animals.	Pearson Correlation	*r* = –.42	*r* = –.45 [–.69, –.12]	Signal-consistent

1b	Mind attribution is positively associated with negative affect regarding eating animals.	Pearson Correlation	*r* = .77	*r* = .80 [.62, .90]	Signal-consistent

1c	Mind attribution is positively associated with moral concern for animals.	Pearson Correlation	*r* = .80	*r* = .83 [.68, .91]	Signal-consistent

2	Being told that animals will be raised for meat consumption (compared to being told they will live as grazing animals) leads to denial of mind for those animals.	Paired Samples *t*-Test	*d* = 0.40	*d* = 0.30 [.24, .37]	Signal-inconsistent, smaller


The meat paradox (Loughnan et al., 2010) has framed much of the psychological approach to meat consumption and has paved the way for the study of human-animal relations to be part of mainstream psychological science (Bastian & Loughnan, 2017; Bratanova et al., 2011; Buttlar & Walther, 2018; Camilleri et al., 2020; Dhont et al., 2021; Dowsett et al., 2018; Graça et al., 2016; Haslam & Loughnan, 2014; Kunst & Hohle, 2016; Leach et al., 2021a, 2021b, 2022; Loughnan & Davies, 2020; Loughnan et al., 2010; Piazza, 2020; Piazza & Loughnan, 2016; Piazza et al., 2015; Rothgerber, 2014). The idea that people are motivated to deny the minds of the animals they eat has intuitive appeal as an explanation of how we can care for animals and simultaneously eat them. However, the replication crisis has clearly shown that the influential nature or intuitive appeal of a psychological account is not always an accurate indicator of its veracity (Zwaan et al., 2018). The findings of this successful replication contribute to the literature on human-animal relations by increasing confidence in the reliability of one of its seminal findings. In short, the work provides further empirical support for the view that motivated mind denial can be a mechanism for resolving the meat paradox.

### Implications, Limitations, and Future Directions

In the target article’s Study 2, Bastian et al. (2012) included a five-minute filler task between judgments of the nonfood animals’ and the food animals’ minds. We decided not to include a filler task, and participants in the present replication provided their perceptions of the food animals’ minds directly following those about nonfood animals’. Presumably, the lack of a filler task would have made any discrepancy in how they reported their perceptions of the animals more noticeable (see e.g., Horne et al., 2015; 2021). We nevertheless found support for the target’s findings regardless, with support for the predicted differences in how participants viewed the minds of food and nonfood animals. The fact that we observed motivated mind denial in such a paradigm either speaks to the strength of the drive for moral disengagement in the face of the meat paradox or to the irrelevance of filler tasks in such paradigms.

There are some societal changes regarding the treatment of animals and consideration of their minds. Plant-based diets seem to have become more common in some parts of the world (Ipsos Retail Performance, 2016), and the popularity of books such as *Are We Smart Enough to Know How Smart Animals Are?* and *Mama’s Last Hug* (de Waal, 2016, 2019) hints at a changing appreciation regarding the sophistication of animal minds. However, in our data on a sample of meat eaters, the endorsement of such views is often barely above the scale midpoint, suggesting that there are still challenges that limit the appreciation of animals’ mental sophistication. This further speaks to the importance of research on this topic, such as the meat paradox, which seeks to understand when and why we fail to see the minds of other animals.

Our approach of conducting a direct replication of Bastian et al. (2012) leaves ample room for future extensions. One area that could be fruitful to explore is how those who do not eat meat respond to similar paradigms. Indeed, Bastian et al. (2012) noted that they investigated this in a pilot study. The meat paradox account predicts that vegetarians and vegans should not show the same inconsistencies in how they attribute minds to food and nonfood animals (Study 2), given that these individuals should not experience the same tension that drives meat eaters to deny food animal minds. Vegetarians and vegans may even be motivated to show the opposite pattern of mind attribution as a way of acknowledging the moral standing of animals who are exploited (Leach et al., 2023a; see also Leach et al., 2023b). Another route that calls for extension is to explore the role of culture. For example, the Australian sample from Bastian et al. (2012) seemed to rate kangaroos as more edible than our American sample, suggesting that culture may influence perceptions of edibility. Work is beginning to test how the meat paradox plays out in other cultures, but data is lacking (see e.g., Tian et al., 2021). Finally, because our work did not attempt to replicate Study 3 of Bastian et al. (2012), future work may aim to replicate it to test the hypotheses that were unique to that study.

Although the current work successfully replicated the results of Bastian et al. (2012) Studies 1 and 2, the close nature of replication means that any limitations from the original studies are likely to be present in the current work. Readers may be concerned about response biases (Wetzel et al., 2013) or common method variance (Lindell & Whitney, 2001) accounting for the results of Study 1. Such factors might produce spurious relationships between constructs at the level of the participant. However, these ought to be minimized when aggregating data to a higher level, as we have done here when collapsing scores to the level of the animal. Given this, it seems unlikely that response biases or common method variance issues could account for the observed relationships. Another aspect that warrants discussion is the selection of the animals in Study 1. How one approaches this does seem to affect the observed relationships. Possidónio et al. (2019) did not observe an association between edibility and perceptions of mind when sampling an overabundance of mammals and birds. This implies that the present data cannot be generalized to all sets of animals. That said, we maintain that the results of Study 1 nevertheless capture an important aspect of how people think about a relevant and important set of animals. They feature a range of groups (mammals, birds, fish, crustaceans, amphibians, reptiles, mollusks, and insects) and include many of the most salient animals to the English language (13 of the 20 most frequently mentioned in the Google Ngram Corpus; Lin et al., 2012). Future work may wish to systematically explore how the findings vary when selecting different groups of animals.

## Conclusion

We successfully replicated a set of seminal findings supporting the idea that people are motivated to deny the minds of the animals they eat (Studies 1 and 2, Bastian et al., 2012). The work contributes by increasing confidence in the reliability of these findings in light of expanding research on how people resolve the meat paradox.

## Additional File

The additional file for this article can be found as follows:

10.5334/irsp.932.s1 Supplementary Materials. Supplementary for Bastian et al. (2012) Replication Registered Report.

## Author Information

Tyler P. Jacobs is a visiting assistant professor at the Swarthmore College Department of Psychology. His work focuses on how self-concept can be leveraged to promote pro-environmental behavior.

Meiying Wang is a Ph.D. student at the Department of Marketing, London Business School, UK. Her work focuses on consumer decision-making.

Stefan Leach is a lecturer in social psychology at Lancaster University, UK. His work focuses on the psychology of meat eating, speciesism, and the moral circle, with a particular emphasis on the role that memory and language play in these processes.

Ho Loong Siu, Mahika Khanna, Ka Wan Chan, and Ho Ting Chau were students at the University of Hong Kong during the academic year 2021/22.

Katy Y. Y. Tam was a Ph.D. candidate at the University of Hong Kong and King’s College London during the academic year 2021/22.

Gilad Feldman is an assistant professor in the University of Hong Kong psychology department. His research focuses on judgment and decision-making.
