# Supplementary material for: Revisiting the Motivated Denial of Mind to Animals Used for Food: Replication Registered Report of Bastian et al. ()
Source: Int Rev Soc Psychol. 2024 Apr 26;37:6. doi: 10.5334/irsp.932 (PMC12372778; doi:10.5334/irsp.932)
Supplement: Supplementary Materials. — Supplementary for Bastian et al. (2012) Replication Registered Report. [file irsp-37-932-s1.pdf]

Revisiting the motivated denial of mind to animals used for food: Replication Registered Report of Bastian et al. (2012)

Supplementary

Contents

Open Science disclosures ... 3

Data collection ... 3

Conditions reporting ... 3

Variables reporting ... 3

Analysis of the original article ... 4

Original article methods ... 4

Experimental design ... 4

Original article results ... 8

Sample size before and after exclusions ... 8

Statistical Results ... 9

Effect size calculations of the original study effects ... 11

Power analysis of original study effect to assess required sample for replication ... 14

Study 1 ... 15

Study 2 ... 17

Materials and scales used in the replication ... 20

Procedure ... 20

Instructions and experimental material ... 22

Scales used in the experiments ... 27

Study 1 ... 27

Study 2 ... 28

Exclusion criteria ... 29

Generalized exclusion criteria ... 29

Specific criteria (exploratory) ... 29

Pre-registration plan versus final report ... 30

Additional analyses and results ... 31

Additional information about the study 34
Replication evaluation 35
    Replication closeness 35
    Replication versus the original 36
References 37

Open Science disclosures

Data collection

Data collection was completed before analyzing the data.

Conditions reporting

All collected conditions are reported.

Variables reporting

All variables collected for this study are reported and included in the provided data.

Analysis of the original article

Original article methods

Experimental design

A summary of type of study, variables tested, and analysis methods is provided in Table S1.

Table S1
Summary of Study 1 and 2 experimental design

Study	Type of study	Independent Variables (IV)	Dependent Variables (DV)	Correlational Variables	Analysis
1	Correlational	-	-	1) Perceived animal mental capacities<br>2) animal edibility<br>3) negative affect<br>4) moral concern	Pearson correlations
2	Within-Subject Experiment	Animal use condition<br>*2 levels: Food vs. Nonfood*	Perceived animal mental capabilities	-	Paired-samples t test*

* It was reported in the manuscript that the independent samples t test was used for analysis. However, we confirmed that this is a description error via personal correspondence with the first author that the paired samples t test was performed in accordance with the with-in subject design.

Study 1

Study 1 was a correlational study. which tested the relationships between animals' perceived mental capacities and their edibility, moral concern about eating them, and negative affect associated with the animal's consumption.

Participants were asked to complete a questionnaire that required them to rate 32 animals. The 32 animals (listed in Table S2) were chosen in a manner to cover a range of wild animals that varied in the extent that they are readily eaten. The participants rate for each animal on the following questions successively using a 1-7 scale: 1) the degree to which each animal possessed 10 mental capacities, 2) the edibility of each animal (2 items), 3) How bad they would feel if they ate each animal, 4) how morally wrong it would be to eat each animal, and finally, 5) whether they were meat eaters. Details about the items for each variable are summarized in Table S3. The text for the mental capacity, negative affect, moral concern, and the vegetarianism question descriptions were inferred based on the Bastian et al. (2012) Study 1 Method section because the exact description was not quoted in the article, whereas the edibility questions were taken verbatim from the article as the items were included there in quotes.

Table S2
Animals chosen for the experiment

**Animal groups**	***N***	**Animal**
Mammals	20	Rabbit, Goat, Dolphin, Deer, Cow, Horse, Sheep, Monkey, Gorilla, Cat, Elephant, Rat, Squirrel, Sloth, Antelope, Wolf, Kangaroo, Lion, Dog, Mole
Birds	3	Sparrow, Pigeon, Chicken
Crustaceans	3	Crab, Prawn, Lobster
Fish	2	Shark, Fish
Amphibians	1	Frog
Reptiles	1	Turtle
Mollusks	1	Snail
Insects	1	Housefly

Note. From Bastian et al. (2012), they focused mostly on mammals for they are generally considered the most similar to humans in mental capacities

Table S3

Details of Measures in Study 1 in the Original

Variables	Item
Mental Capacity	To which degree does (animal) possess the following mental capacities? (on a 7-point scale; 1 = definitely not possess, 7 = definitely does not possess)<br><br>10 mental capacities: hunger, fear, pleasure, pain, rage, self-control, morality, memory, emotion recognition, planning
Edibility	1) Would you choose to eat this animal? (on a 7-point scale; 1 = definitely would not, 7 = definitely would)<br>2) Would you eat this animal if asked to? (on a 7-point scale; 1 = definitely would not, 7 = definitely would)
Negative affect	How bad would you feel if you ate each animal? (on a 7-point scale; 1 = not at all, 7 = extremely)
Moral perception	How morally wrong would it be to eat each animal? (on a 7-point scale; 1 = not at all, 7 = extremely)
Vegetarian or Vegan (Exclusion criteria)	Are you a vegetarian?<br>(indicating either yes or no)

Study 2

Study 2 implemented a within-subjects (Food Condition vs. Nonfood Condition) experimental design. All participants went through the nonfood condition first, then completed a 5-min unrelated task, and then went through the food condition. In the nonfood condition, a picture of a lamb or a cow surrounded by green grass (see Figure 1) was presented and the animal was described as being bred for meat consumption. In the food condition, a picture of a cow or a lamb surrounded by green grass was presented and the animal was described as living on a farm. Participants were randomly assigned to complete one of the two versions of the questionnaire: the picture of lamb was presented first as the nonfood animal and the picture of cow was presented at last as the food animal in one version, while the picture of cow was presented first as the nonfood animal and the picture of lamb was presented at last as the food animal in the other version. After reading the descriptions and looking at the picture, participants in both conditions rated the animal on 15 mental capacities (see Table 4 of the manuscript).

Figure S1.
Pictures used in study 2.

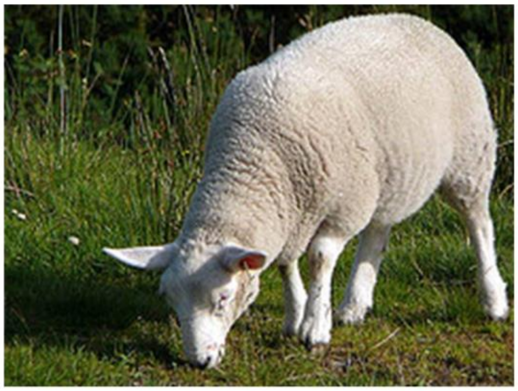
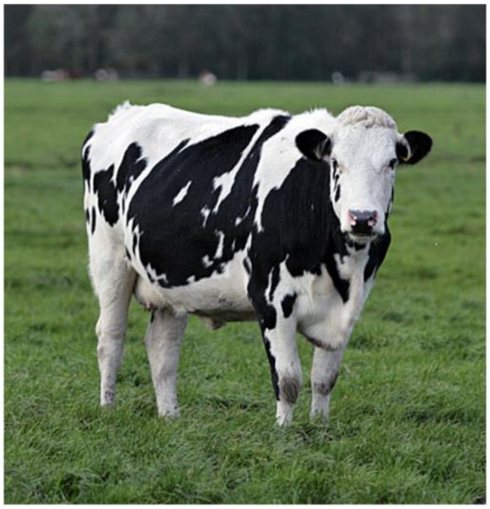

Note. Pictures were directly acquired from correspondence with the first author of Bastian et al. (2012).

Original article results

Sample size before and after exclusions

In the original article, study 1 had a sample size of 71 before exclusion and 63 after exclusion. Participants who identified as vegetarians or vegans were excluded from analysis. In study 2, the sample size was 66 and no exclusion criteria was reported. Table S4 summarizes the sample descriptions of Study 1 & 2 in the original article.

Table S4

Sample descriptions of Study 1 & 2 in the original article

	**Study 1**	**Study 2**
Sample size (before exclusion)	71	66
Sample size (after exclusion)	63	66
Exclusion	Vegetarians; Reason not specified	Not reported
Age	Range: 17 - 29, *M* = 19.13, *SD* not specified	Range: 17 - 52, *M* = 19.23, *SD* not specified
Gender	59 Female; 12 Male (Before exclusion; after exclusion not specified)	43 Female; 21 Male
Location	Recruited from a large Australian university	Recruited from a large Australian university
Sample type	Students	Students
Other details	Participants recruited were 1st-year students	Participants recruited are meat-eaters. Meat eaters were identified by self-report.

Statistical Results

Study 1

Principal component analyses for the 10 mental capacities conducted for each animal revealed that all mental capacities loaded onto one factor that explained 29% to 48% of the variance.

Table S5 summarizes the statistical results for the correlational tests in Study 1.

Table S5

Statistical results of Study 1

Variables		*N*	*df*	*r*	*95%CI*	*p*
Perceived Mind	Animal Edibility	32	30	-.42	[-.67, -.08]	<.001
	Negative Affect	32	30	.77	[.58, .88]	<.001
	Moral concerns	32	30	.80	[.63, .90]	<.001

Note. The degree of freedom and 95%CI for the effect sizes were not reported in the original article. An earlier version (Stage 1) misspecified the sample size as 71 participants rather than 32 animals.

Study 2

It was reported in the manuscript that the independent samples t-test was used for analysis (see highlighted section in Figure 2). However, we confirmed that this is a description error via personal correspondence with the first author, who confirmed that a paired samples t-test was performed in accordance with the within subject design.

Figure S2

Screenshot of Study 2 results part in Bastian et al. (2012, pp.250)

Results

Mean mental capacity ratings were calculated for each animal and each condition. Participants' ratings of sheep and cows did not differ within either condition so we collapsed across versions. This yielded two animal types: food animal and nonfood animal. An independent samples *t* test indicated that when reminded that an animal would be used for food, meat eaters denied it mental capacities (food animal: $M = 4.08$, $SD = .86$) compared to when no such reminders where provided (nonfood animal: $M = 4.30$, $SD = .82$), $t(65) = 3.24$, $p = .002$ (see Figure 2).[1]

Results from the paired samples t-test examining the effect of food use descriptions on animals' perceived mental capacities are summarized in Table S6.

Table S6

Descriptives and statistical test results of animals' perceived mental capacities by condition

Animal Use Condition	*N*	*M*	*SD*	*t*	Cohen's *d*	*95% CI*	*p*
Food Condition	66	4.08	0.86	3.24	0.40	[0.15, 0.65]	.002
Nonfood Condition	66	4.30	0.82				

<u>Effect size calculations of the original study effects</u>

R markdown file and output HTML file for the following calculations are available on OSF (“ES-calculation-and-power-analysis.Rmd/html” and “Sensitivity Analysis.Rmd/html).

Study 1

1. Effect size with 95%CI for the correlation between perceived mental capacity and animal edibility generated using MBESS. NOTE: An earlier version (Stage 1) misspecified the sample size as 71 participants rather than 32 animals.

Input:

```
> ci.cc(r=-.42, n=32, conf.level = .95)
```

Output:

```
## $Lower.Limit
## [1] -0.6704985
##
## $Estimated.Correlation
## [1] -0.42
##
## $Upper.Limit
## [1] -0.08354069
```

2. Effect size with 95%CI for the correlation between perceived mental capacity and feeling bad about eating the animal using MBESS.

Input:

```
> ci.cc(r=.77, n=32, conf.level = .95)
```

Output:

```
## $Lower.Limit
## [1] 0.5759436
##
## $Estimated.Correlation
## [1] 0.77
##
## $Upper.Limit
```

```
## [1] 0.8819069
```

3. Effect size with 95%CI for the correlation between perceived mental capacity and moral concern about eating the animal using MBESS.

Input:

```
> ci.cc(r=.80, n=32, conf.level = .95)
```

Output:

```
## $Lower.Limit
## [1] 0.6259057
##
## $Estimated.Correlation
## [1] 0.8
##
## $Upper.Limit
## [1] 0.8981503
```

Study 2

1. Effect size and 95% CI for paired samples t test on mental attribution ratings by food/ nonfood animal groups using MBESS

Input:

```
> ci.sm(ncp = 3.24, N = 66)
```

Output:

```
## [1] "The 0.95 confidence limits for the standardized mean are given as:"
## $Lower.Conf.Limit.Standardized.Mean
## [1] 0.1466243
##
## $Standardized.Mean
## [1] 0.3988164
##
## $Upper.Conf.Limit.Standardized.Mean
## [1] 0.6481647
```

Power analysis of original study effect to assess required sample for replication

The required sample size for 0.95 power and 0.05 alpha in Study 1 is 448, while that in Study 2 is 157. Combined together, the final sample size required for the replication is 448. To account for deviations from the original article and possible exclusions, we would like to aim for a sample size of 800 for data collection. The effect size calculations and power analysis results for each effect in study 1 and 2 are summarized in Table S7.

Table S7

Summary of the effect size, 60% CIs, and required sample size of the original article

	r	***60%CIL***	***60%CIH***	***d***	***Required sample size (N)***
Hypothesis 1a (perceived mind negatively correlated with animal edibility)	-.42	-0.500	-0.332	-	**112 x 4 = 448**
Hypothesis 1b (perceived mind positively correlated with feeling bad)	.77	0.725	0.808	-	18
Hypothesis 1c (perceived mind positively correlated with morally wrong)	.80	0.760	0.834	-	16
Hypothesis 2 (Significant difference between food condition and non-food condition)	-	0.290	0.505	0.399	157

Note. Results are rounded off in 3 decimal places. The lower 60% confidence interval for the original effect size is the safeguard effect size passed on for power analysis.

Below are the power analysis calculations for all the effects in study 1 and 2 using packages <pwr> <MOTE> and <MBESS> in R. R markdown file and output HTML file for the following calculations are available on OSF (“ES-calculation-and-power-analysis.Rmd/html”).

We used the safeguard power analysis method (Perugini et al., 2014) to conservatively estimate the sample size needed for replication.

Step 1: Calculate the 60% CIs for the effect sizes above.

Step 2: Calculate required sample size using the lower bound of the 60% CIs as effect size, targeting 95% power.

Study 1

The estimated sample size required for study 1 is 112. However, since we have made modification in our replication so that each participant will only be required to rate 8 animals instead of 32, the required sample size is thus multiplied by 4 resulting in a total sample size of 448.

1. Correlation between perceived mental capacity and animal edibility

Input:

```
#Estimate 60% CI for r from Bastian et al (Study 1, correlation between perceived mind and edibility)
ci.cc(r=-.42, n=71, conf.level = .60)
#Required sample size to detect safeguard r = -.332 at 95% power
pwr.r.test(r=-.332, sig.level = .05, power = .95)
```

Output:

```
## $Lower.Limit
## [1] -0.5003355
##
## $Estimated.Correlation
## [1] -0.42
##
## $Upper.Limit
## [1] -0.3324947

##
##      approximate correlation power calculation (arctangh transformation)
##
##               n = 111.2568
```

```
##              r = 0.332
##      sig.level = 0.05
##          power = 0.95
##    alternative = two.sided
```

2. Correlation between perceived mental capacity and feeling bad about eating the animal

Input:

```
#Estimate 60% CI for r from Bastian et al (Study 1, correlation between perceived mind
and feeling bad about eating the animal)
ci.cc(r=.77, n=71, conf.level = .60)
#Required sample size to detect safeguard r = .725 at 95% power
pwr.r.test(r=.725, sig.level = .05, power = .95)
```

Output:

```
 ## $Lower.Limit
## [1] 0.7250762
##
## $Estimated.Correlation
## [1] 0.77
##
## $Upper.Limit
## [1] 0.8083984

##
##     approximate correlation power calculation (arctangh transformation)
##
##              n = 17.77299
##              r = 0.725
##      sig.level = 0.05
##          power = 0.95
##    alternative = two.sided
```

3. Correlation between perceived mental capacity and moral concern about eating the animal

Input:

```
#Estimate 60% CI for r from Bastian et al (Study 1, correlation between perceived mind and morality)
ci.cc(r=.80, n=71, conf.level = .60)
#Required sample size to detect safeguard r = .760 at 95% power
pwr.r.test(r=.760, sig.level = .05, power = .95)
```

Output:

```
 ## $Lower.Limit
## [1] 0.7601417
##
## $Estimated.Correlation
## [1] 0.8
##
## $Upper.Limit
## [1] 0.83386

##
##     approximate correlation power calculation (arctangh transformation)
##
##               n = 15.48467
##               r = 0.76
##       sig.level = 0.05
##           power = 0.95
##     alternative = two.sided
```

<u>Study 2</u>

1. Effect of Animal use conditions on perceived mental capacities

Input:

```
#Estimate 60% CI for dz from Bastian et al (Study 2, 2012)
d.dep.t.diff.t(t = 3.24,
               n = 66,
               a = 0.40) #setting alpha to 0.40 gives the 60% CI

#Required sample size to detect safeguard dz = 0.290 at 95% power
pwr.t.test(d = 0.290,
           power = 0.95,
           sig.level = 0.05,
           type = "paired",
           alternative = "two.sided")
```

Output:

```
## $d
## [1] 0.3988164
##
## $dlow
## [1] 0.2895919
##
## $dhigh
## [1] 0.5049567
##
## $n
## [1] 66
##
## $df
## [1] 65
##
## $t
## [1] 3.24
##
## $p
## [1] 0.001885543
##
## $estimate
## [1] "$d_z$ = 0.40, 60\\% CI [0.29, 0.50]"
##
```

```
## $statistic
## [1] "$t$(65) = 3.24, $p$ = .002"

##
##      Paired t test power calculation
##
##               n = 156.4498
##               d = 0.29
##       sig.level = 0.05
##           power = 0.95
##     alternative = two.sided
##
## NOTE: n is number of *pairs*
```

Sensitivity analysis of original study effect to assess required sample for replication

Sensitivity Analysis for Bastian et al. (2012) Study 1 Replication

```
library(pwr)
pwr.r.test(n = 1000, sig.level = .05, power = 0.95)

##
##      approximate correlation power calculation (arctangh transformation)
##
##               n = 1000
##               r = 0.1136188
##       sig.level = 0.05
##           power = 0.95
##     alternative = two.sided
```

Sensitivity Analysis for Bastian et al. (2012) Study 2 Replication

```
library(pwr)

pwr.t.test(n= 1000, sig.level = .05, power = 0.95,
           type = "paired")

##
##      Paired t test power calculation
##
##               n = 1000
##               d = 0.1141078
##       sig.level = 0.05
##           power = 0.95
##     alternative = two.sided
##
## NOTE: n is number of *pairs*
```

Materials and scales used in the replication

Procedure

Participants first read and agreed with the consent form. They then read about the study outline with questions asking whether they would commit to pay attention, informing them of manipulation and attention checks, and whether they were native English speakers born and raised in the US. Participants then entered either study 1 or 2, which order was randomized and counterbalanced.

In study 1, participants rated 8 animals randomly selected out of 32 on 4 consecutive pages. For each animal, they indicated their perceived mental capacity for the animal on 10 items using a 1 to 7 scale. Right below these ratings, they were asked 4 questions to indicate their opinions on the edibility of the animal, how bad they would feel about eating the animal, and how morally wrong it would be to eat the animal.

In study 2, participants were randomly assigned to one of the two versions: in version 1, they first saw a picture of a lamb surrounded by green grass described as a nonfood animal, and later on another page they saw a picture of a cow surrounded by green grass described as a food animal. In version 2, the cow showed up first as a nonfood animal, and the lamb showed up later as a food animal. Then, participants once again saw the picture and the caption and were asked to rate 15 mental capacities for the animal using a 1 to 7 scale. After completing the scale, participants answered a manipulation check asking about the fate of the animal. B were Table S8 summarizes the procedure for study 2.

After the main tasks, participants went through 2 attention check questions and 4 funneling questions about the survey, 6 demographics questions, 1 question about their satisfaction with the payment, and finally read debriefing information about the survey before exiting.

All materials, scales, and instructions can be found in the next two sections (Instructions and experimental material and Scales used in the experiments) of the supplementary.

Table S8
Summary of Study 2 Procedure

Qualtrics version	Version 1 (Cow as control)	Version 2 (Lamb as control)
**Scenario 1** (Nonfood condition)	Cow	Lamb
Picture shown	A picture of Cow living on a farm, surrounded by grass (see Figure 1)	A picture of Lamb living on a farm, surrounded by grass (see Figure 1)
Description shown	“This cow will be moved to other paddocks, and will spend most of its time eating grass with other cows”	“This lamb will be moved to other paddocks, and will spend most of its time eating grass with other lamb”
Mental Capacities Rating (15 items: *pleasure, fear, rage, joy, happiness, desires, wishes, planning, goals, pride, pain, hunger, tasting, seeing, hearing*)	“To what extent does this cow possess the following mental capacities?” (on a 7-point scale; 1 = *definitely does not possess*, 7 = *definitely does possess*)	“To what extent does this lamb possess the following mental capacities?” (on a 7-point scale; 1 = *definitely does not possess*, 7 = *definitely does possess*)
	(Next page)	(Next page)
Manipulation check	“To make sure that you read and understood the scenario, in the last scenario, what was the fate of the animal??” 1 = It was sent to paddocks to eat grass with other animals, 2= It was butchered and treated as a meat product 3 = It was released to live in a forest	“To make sure that you’ve read and understood the scenario, in the last scenario, what was the fate of the animal??” 1 = It was sent to paddocks to eat grass with other animals 2= It was butchered and treated as a meat product 3 = It was released to live in a forest
**Scenario 2** (Food condition)	Lamb	Cow
Picture shown	A picture of lamb living on a farm, surrounded by grass	A picture of Cow living on a farm, surrounded by grass

Caption shown	"This lamb will be moved to other paddocks, and will spend most of its time eating grass with other cows"	"This cow will be taken to an abattoir, killed, butchered, and sent to supermarkets as meat products for humans"
Mental Capacities Rating (15 items: *pleasure, fear, rage, joy, happiness, desires, wishes, planning, goals, pride, pain, hunger, tasting, seeing, hearing*)	"To what extent does this lamb possess the following mental capacities?" (on a 7-point scale; 1 = *definitely does not possess*, 7 = *definitely does possess*)	"To what extent does this cow possess the following mental capacities?" (on a 7-point scale; 1 = *definitely does not possess*, 7 = *definitely does possess*)
	(Next page)	(Next page)
Manipulation check	"To make sure that you read and understood the scenario, in the last scenario, what was the fate of the animal??"<br>1 = It was sent to paddocks to eat grass with other animals,<br>2= It was butchered and treated as a meat product<br>3 = It was released to live in a forest	"To make sure that you read and understood the scenario, in the last scenario, what was the fate of the animal??"<br>1 = It was sent to paddocks to eat grass with other animals,<br>2= It was butchered and treated as a meat product<br>3 = It was released to live in a forest

Instructions and experimental material

All participants first read the consent form and confirmed that they were 18 years old or older and agreed to continue with the study.

The survey includes a page introducing the study outline and 3 verification questions at the beginning, which includes a reminder for the comprehension check:

(outline1) Are you able to pay close attention to the details provided and carefully answer questions that follow?

o Yes (1)

o No (0)

o Not sure, probably not (99)

(outline2) = WARNING: Survey includes attention and comprehension checks. If you do not like participating in surveys with checks, please return the HIT now.=

Do you understand the study outline and are willing to participate in a survey with comprehension checks?

- o Yes (1)
- o No (0)
- o Not sure, probably not (99)

(native) This survey is only intended for native English speakers born and raised in the United States.

Are you a native English speaker born, raised, and currently located in the US?

- o Yes (1)
- o No (0)

Study 1

For each animal, participants rated the animal's 10 mental capacities on a 1-7 scale. Take the [rabbit] animal as an example:

RabbitMental

To which degree does ***a rabbit*** possess the following mental capacities? (On a 7-point Scale: 1= definitely does not possess , 7=definitely does possess)

	1 (Definitely does not possess)	2	3	4	5	6	7 (Definitely does possess)
Hunger	○	○	○	○	○	○	○
Fear	○	○	○	○	○	○	○
Pleasure	○	○	○	○	○	○	○
Pain	○	○	○	○	○	○	○
Rage	○	○	○	○	○	○	○
Self-control	○	○	○	○	○	○	○
Morality	○	○	○	○	○	○	○
Memory	○	○	○	○	○	○	○
Emotion recognition	○	○	○	○	○	○	○
Planning	○	○	○	○	○	○	○

Participants then answer 4 more questions about edibility, negative affect, and moral concern with the following instruction:

Please answer the following questions on a 7-point scale, with 1= "Definitely would not" and 7= "Definitely would".

Study 2

Experimental condition: Nonfood condition

Version 1: Cow as nonfood animal

Please look carefully at this cow in the picture.

This cow will be moved to other paddocks, and will spend most of its time eating grass with other cows.

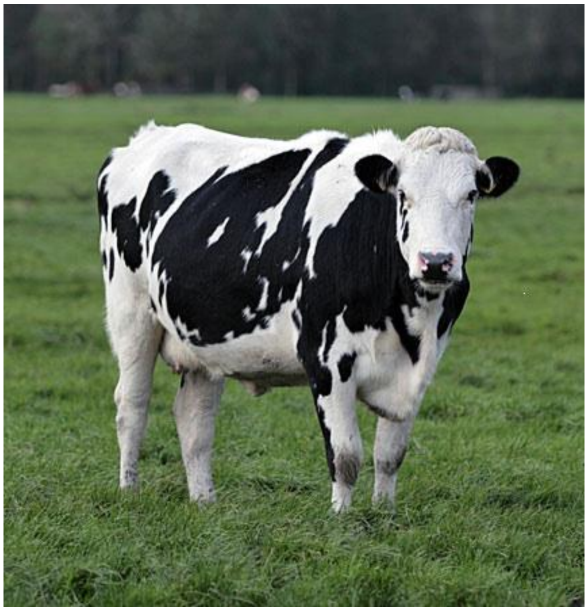

Version 2: Lamb as nonfood animal

Please look carefully at this lamb in the picture.

This lamb will be moved to other paddocks, and will spend most of its time eating grass with other lambs.

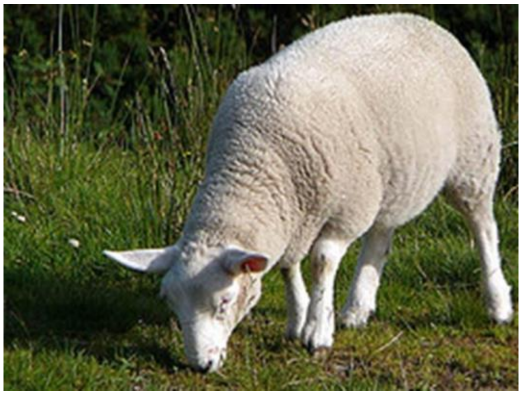

Experimental condition: Food condition

Version 1: Lamb as food animal

Please look carefully at this lamb in the picture.

This lamb will be taken to an abattoir, killed, butchered, and sent to supermarkets as meat products for humans.

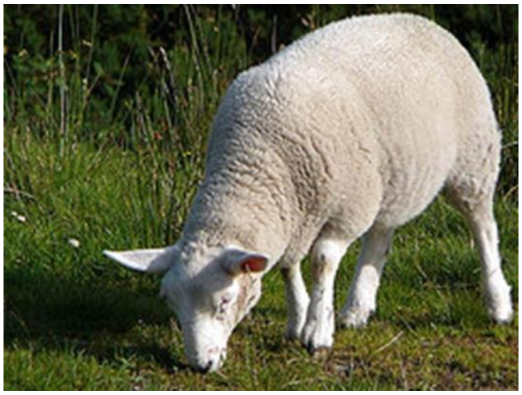

Version 2: cow as food animal

Please look carefully at this cow in the picture.

This cow will be taken to an abattoir, killed, butchered, and sent to supermarkets as meat products for humans.

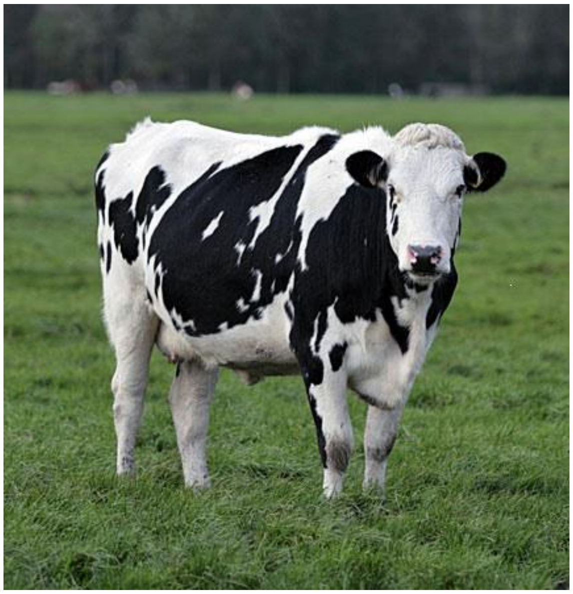

Dependent variables :

To what extent does this [lamb/cow] possess the following mental capacities? (on a 7-point scale; 1 = Definitely does not possess, 7 = Definitely does possess)

15 mental capacities: pleasure, fear, rage, joy, happiness, desires, wishes, planning, goals, pride, pain, hunger, tasting, seeing, hearing

Manipulation check:

To make sure that you read and understood the scenario, in the last scenario, what was the fate of the animal?

1 = It was sent to paddocks to eat grass with other animals

2= It was butchered and treated as a meat product

3 = It was released to live in a forest

Attention check questions

Which of the following have you done within the past week? Please select all that apply:

- ☐ Ran a half-marathon or marathon
- ☐ Purchased a new television
- ☐ Used a computer, tablet, or mobile phone
- ☐ Ran a mile in less than 3 minutes
- ☐ Ate a pre-packaged frozen dinner
- ☐ Read a book
- ☐ Donated to a charity
- ☐ ⃠ None of the above

How serious were you in filling out this questionnaire?

1 - Not at all	2	3	4	5 - Very much
○	○	○	○	○

Meat-Eater Status Question

Do you eat meat?

- ○ Yes, I eat meat.
- ○ No, I do NOT eat meat.

Funneling section

Three funneling questions:

- *What do you think the purpose of the last part was?*
- *Have you ever seen the materials used in this study or similar before? If yes - please indicate where*
- *Did you spot any errors? Anything missing or wrong? Something we should pay attention to in next runs? (Briefly, up to one sentence, write "none" if not relevant)*

Finally, participants were asked to fill in demographics (age, gender, country-of-origin, country-of-origin, and understanding of English) and were debriefed.

<u>Scales used in the experiments</u>

<u>Study 1</u>

1. Mental capacities

“To which degree does (animal) possess the following mental capacities?” for 10 mental capacities (on a 7-point scale; 1 = definitely not possess, 7 = definitely does possess)

10 mental capacities: hunger, fear, pleasure, pain, rage, self-control, morality, memory, memory-recognition, planning

2. Animal’s Edibility

Item 1) “Would you choose to eat this animal?” (on a 7-point scale; 1 = definitely would not, 7 = definitely would)
Item 2) “Would you eat this animal if asked to?” (on a 7-point scale; 1 = definitely would not, 7 = definitely would)

3. Negative Affect

“How bad would you feel if you ate each animal?” (on a 7-point scale; 1 = not at all, 7 = extremely)

4. Moral concern

“How morally wrong would it be to eat each animal?” (on a 7-point scale; 1 = not at all, 7 = extremely)

Study 2

1. Mental capacity

“To what extent does this animal possess the following mental capacities?” for 15 mental capacities on a 7-point Likert scale (1 = definitely does not possess; 7 = definitely does possess)

15 mental capacities: pleasure, fear, rage, joy, happiness, desires, wishes, planning, goals, pride, pain, hunger, tasting, seeing, hearing

Exclusion criteria

Generalized exclusion criteria

We focused on our analyses on the full sample who meet our general criteria for inclusion. However, in the event of a failed replication we would have also excluded participants who failed our exploratory specific criteria.

General criteria:

1. Participants indicating a low proficiency of English (self-report < 5, on a 1-7 scale)
2. Participants who self-report not being serious about filling in the survey (self-report < 4, on a 1-5 scale).
3. Participants who correctly guessed the hypothesis of this study in the funneling section.
4. Participants who have already seen or done the survey before.
5. Participants who failed to complete the survey. (duration = 0 or have left questions blank)
6. Participants not from the US.
7. Participants aged below 18. [there is a type-in answer box about participants' age in Qualtrics template with default age range 1-100]
8. As this research focuses on the effect of meat paradox on the mind perception of meat-eaters, we excluded all non meat-eaters from the study. Although Prolific recruitment filters will be used to only recruit meat-eaters, an item indicating whether the participant was a meat eater was included at the end of the questionnaire.

Specific criteria (exploratory)

1. In the event of a failed replication, participants who failed either of the attention checks or the manipulation check would have been excluded.

Pre-registration plan versus final report

See Preregistration Planning and Deviation Documentation (PPDD) document for latest updates.

Components in preregistration	Location of 1) preregistered decision/plan and 2) rational for decision/plan	Were there deviations? What type?	If yes - describe details of deviation(s)	Rationale for deviation	How might the results be different if you had/had not deviated	Date/time of decision for deviation + stage
Study design		No				
Measured variables		No				
Exclusion criteria	Supplemental Materials, pg. 28	Minor	In the Supplemental Materials, clarified which criteria were used to be consistent with the main manuscript	To be consistent with main manuscript and our preregistration	The results do not change in terms of LeBel's criteria if different exclusions are used.	August 8th, 11:43AM
IV		No				
DV		No				
Data analysis	Results section	Minor	Added in animal level analyses in Study 1 and moved the participant-level analyses to the Supplementals	The animal level analyses were specified in the methods and were what was performed in the original article	The correct analyses for Study 1 would be missing	July 17, 4:39PM

Note. *Categories for deviations: Minor - Change probably did not affect results or interpretations; Major - Change likely affected results or interpretations.

Additional analyses and results

We explored the potential effect of the animal species (cow vs. lamb) by conducting a 2 x 2 mixed factorial ANOVA with animal food status (food vs. nonfood) as the within-subjects factor and animal species (cow-first vs. lamb-first) as the between-subjects factor and mind attribution as the dependent variable. Cows were attributed no more mind than lambs,(F(1, 957) = 0.26, p = .612, η_p^2 = < .001, 90% CI [.00, .005]). There was also no reason to believe that the animal species moderated the differences in mind attribution to food animals compared to nonfood animals, F(1, 957) = 0.97, p = .324, η_p^2 = .001, 90% CI [.00, .01].

Next, we calculated Pearson correlations between the variables in Study 1 and the dependent variables in Study 2. We conducted this analysis as a measure of whether completing both studies in the same session was leading to the responses converging in a way that would not otherwise occur. As seen in Table S9, there were significant correlations between Study 1 perceived animal mental capacities and Study 2 perceived mental capacities both food or nonfood animals and these effects were large. There were no significant correlations between Study 1 perceived animal edibility and Study 2 perceived mental capacities for food or nonfood animals and these effects were very small. There were significant positive correlations between Study 1 negative affect and Study 2 perceived mental capacities for food or nonfood animals and these effects were small. Finally, there were significant positive correlations between Study 1 moral concern and Study 2 perceived mental capacities for food or nonfood animals and these effects were small.

Table S10

Pearson correlations between Study 1 and Study 2 Variables

		Study 2	
		Food Condition Capacities	Nonfood Condition Capacities
Study 1			
Mental Capacities			
	r	.74	.75
	p-value	< .001	< .001
	95% CI	[.71, .77]	[.72, .78]
Animal Edibility			
	r	-.004	-.03
	p-value	.894	.316
	95% CI	[-.07, .06]	[-.10, .03]
Negative Affect			
	r	.14	.16
	p-value	< .001	< .001
	95% CI	[.08, .21]	[.11, .23]
Moral Concern			
	r	.10	.11
	p-value	.002	< .001
	95% CI	[.03, .16]	[.05, .18]

We also ran a set of additional analyses to test if the order of two studies moderated any participant-level effects. First, in Study 1, there was no interaction between study order and perceived animal mental capabilities for perceived animal edibility (β = -0.02, t(955) = -0.27, p = .170, 95% CI [-0.15, 0.11]) and there was no main effect of study order (β = -0.07, t(955) = -1.58, p = .115, 95% CI [-0.15, 0.02]) or perceived animal mental capabilities (β = -0.05, t(955) = -1.11, p = .269, 95% CI [-0.15, 0.04]) on perceived animal edibility.

Second, in Study 1, there was no interaction between study order and perceived animal mental capabilities for negative affect (β = -0.01, t(955) = -0.16, p = .871, 95% CI [-0.14, 0.11]) and there was no main effect of study order (β = 0.02, t(955) = 0.23, p = .817, 95% CI [-0.10, 0.14]). There was a main effect of perceived animal mental capabilities (β = 0.01, t(955) = 0.07, p = .947, 95% CI [-0.08, 0.09]) such that greater perceived mental capabilities predicted greater negative affect when controlling for study order and the interaction.

Third, in Study 1, there was no interaction between study order and perceived animal mental capabilities for moral concern for animals (β = 0.03, t(955) = -0.53, p = .597, 95% CI [-0.16, 0.09]) and there was no main effect of study order (β = -0.05, t(955) = 0.36, p = .716, 95% CI [-0.17, 0.08]). There was a main effect of perceived animal mental capabilities (β = 0.25, t(955) = 5.26, p = < .001, 95% CI [0.16, 0.35]) on moral concern such that greater perceived mental capabilities predicted greater moral concern when controlling for study order and the interaction.

Next, in Study 2, a mixed ANOVA found that there was no interaction between food status of the animal and study order for perceived animal mental capabilities, F(1, 957) = 2.79, p = .095, η_p^2 = .003, 90% CI [.00, .01]. There was still a main effect of food status of the animal (F(1, 957) = 87.11, p < .001, η_p^2 = .08, 90% CI [.05, .11]). There was a main effect of study order (F(1, 957) = 10.65, p = < .001, η_p^2 = .01, 90% CI [.00, .02]) such that participants who completed Study 1 first rated the animals in Study as generally having more mental capabilities. However, because the interaction was not significant, study order did not moderate the effects in this study.

To further explore any impacts of the order of the studies, we reexamined our primary analyses only when a given study was presented first. The animal-level results for Study 1 when presented first can be found in Table S11. We found support for greater perceived mental capacity being associated with less perceived animal's edibility (H1a). We found support for perceived mental capacity being associated with feeling bad about eating animals (H1b). Finally, we found support for perceived animals' mental capacity being associated with how morally wrong it would be to eat the animal (H1c). This is the same pattern of findings as in our original analyses with both study orders included.

Table S11

Study 1 when presented first: Summary of means, standard deviations, and correlations with animal's perceived mental capacities

Variable	*Mean*	*SD*	*r*	*p*	***95% CI Upper***	***95% CI Lower***
Mental Capacities	4.55	0.80	-	-	-	-
Animal Edibility	2.81	1.65	-.49	.004	-.17	-.72
Negative Affect	3.99	1.38	.81	< .001	.90	.65

Moral Concern	3.52	1.24	.84	< .001	.92	.69

Note. N = 1000

We also reexamined the findings of Study 2 when it was presented first. We calculated mean mental capacity ratings for the food (N = 500, M = 4.75, SD = 1.05) and nonfood conditions (M = 4.91, SD = 0.93). We then conducted a paired-samples t-test. There was support for the hypothesis: being informed that an animal would be used for food lead to less mind attribution compared to being informed that an animal would not be used for food, $t(499) = 6.77$, $p < .001$, d = 0.30, 95% CI [0.21, 0.39]. The effect was in the same direction of the hypothesis and was small-to-medium in size, supporting Hypothesis 2. This is the same pattern of findings as in our original analyses with both study orders included.

Additional information about the study

Data collection procedures:

This study was conducted on Prolific with American participants. We imposed the following settings in recruiting our participants:

1. Participants were paid $1.90USD as a fixed participation reward.
2. The expected completion time was set at 10 minutes in advance.
3. Participants were required to complete the study on a desktop computer.
4. We used a sample with a balanced gender distribution.
5. We limited all participants Approval Rate to be between 95% and 100%.
6. We limited each worker's number of previous Prolific submissions to be between 100 and 100,000.
7. We blocked duplicate IP addresses and duplicate geolocation.
8. We restricted participants' location to be in the U.S.
9. We enabled only participants with no dietary restrictions and who reported not following any diet.

Replication evaluation

<u>Replication closeness</u>

Figure S3.

Criteria for evaluation of replications by LeBel et al. (2018)

Target similarity	Highly similar				Highly dissimilar
Category	Direct replication				Conceptual replication
Design facet	**Exact replication**	**Very close replication**	**Close replication**	**Far replication**	**Very far replication**
Effect/hypothesis	Same/similar	Same/similar	Same/similar	Same/similar	Same/similar
IV construct	Same/similar	Same/similar	Same/similar	Same/similar	Different
DV construct	Same/similar	Same/similar	Same/similar	Same/similar	Different
IV operationalization	Same/similar	Same/similar	Same/similar	Different	
DV operationalization	Same/similar	Same/similar	Same/similar	Different	
Population (e.g. age)	Same/similar	Same/similar	Same/similar	Different	
IV stimuli	Same/similar	Same/similar	Different		
DV stimuli	Same/similar	Same/similar	Different		
Procedural details	Same/similar	Different			
Physical setting	Same/similar	Different			
Contextual variables	Different				

Note. A classification of relative methodological similarity of a replication study to an original study. “Same” (“different”) indicates the design facet in question is the same (different) compared to an original study. IV = independent variable. DV = dependent variable. “Everything controllable” indicates design facets over which a researcher has control. Procedural details involve minor experimental particulars (e.g., task instruction wording, font, font size, etc.).

"Similar" category was added to the Lebel et al. (2018) typology to refer to minor deviations or extensions aimed to adjust the study to the target sample that are not expected to have major implications on replication success. See Olsson-Collentine, van Assen, and Wicherts (2020) on meta analysis showing minor to no expected impact due to variations in sample population or setting.

<u>Replication versus the original</u>

Figure S4.
Interpretation criteria for evaluation of replications outcomes by LeBel et al. (2019), if the original study detected a signal

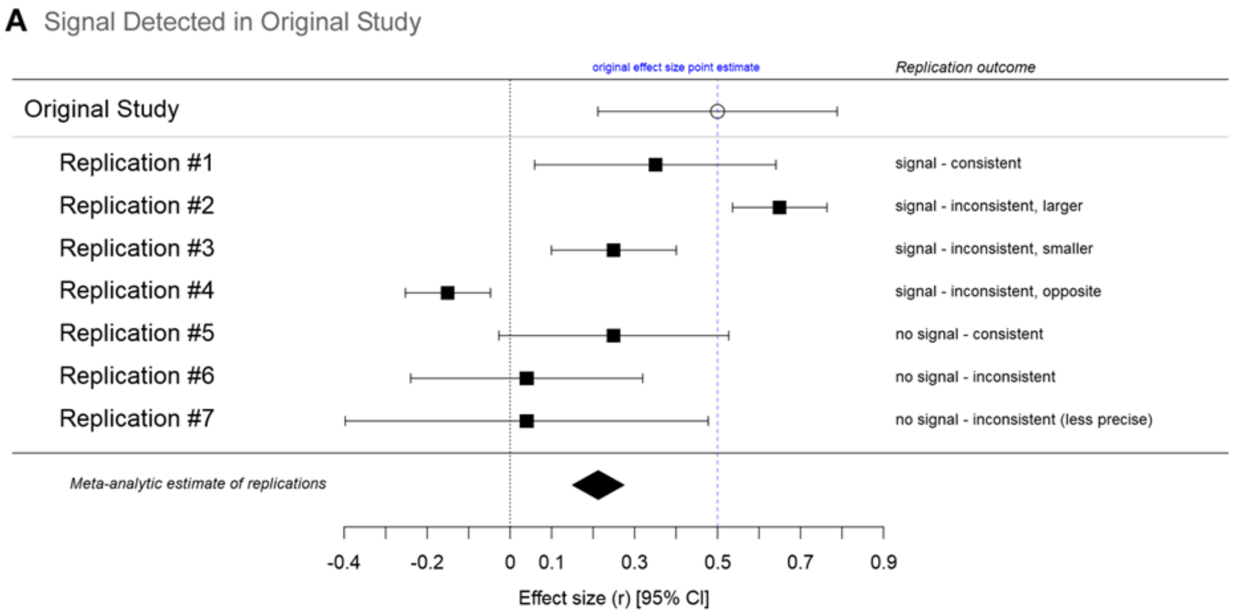

Figure S5
Interpretation criteria for evaluation of replications outcomes by (LeBel et al., 2019), if the original study failed to detect a signal.

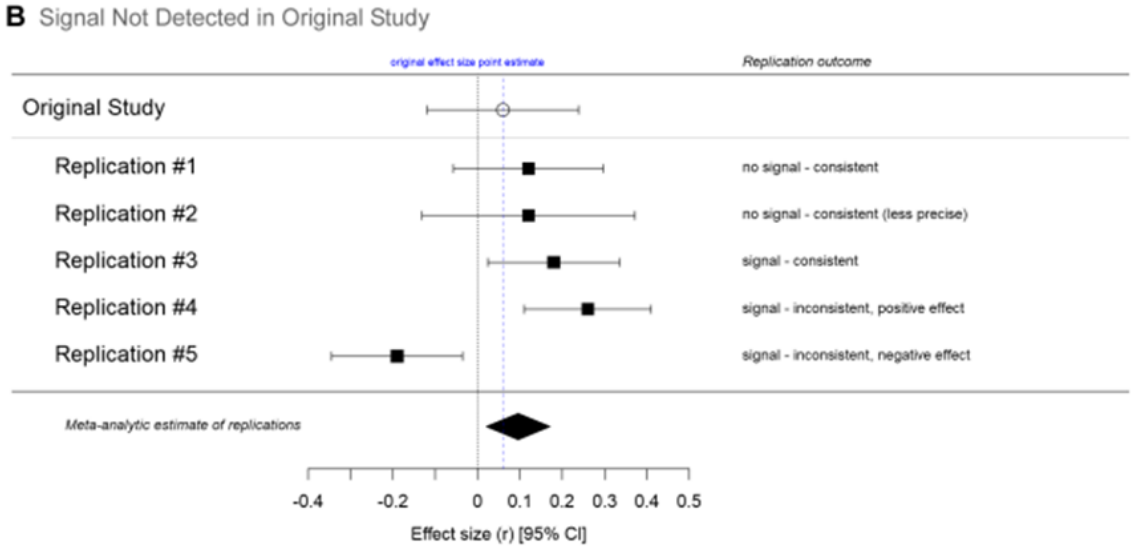

References

Aczel, B., Szollosi, A., & Bago, B. (2018). The effect of transparency on framing effects in within- subject designs. *Journal of Behavioral Decision Making*, 31(1), 25-39.

Ipsos Retail Performance. (2020). Exploring the explosion of veganism in the United States. Retrieved from https://www.ipsos-retailperformance.com/en/vegan-trends/.

LeBel, E. P., McCarthy, R. J., Earp, B. D., Elson, M., & Vanpaemel, W. (2018). A unified framework to quantify the credibility of scientific findings. *Advances in Methods and Practices in Psychological Science*, *1*, 389-402.

LeBel, E. P., Vanpaemel, W., Cheung, I., & Campbell, L. (2019). A brief guide to evaluate replications. *Meta-Psychology*, 3.

Perugini, M., Gallucci, M., & Costantini, G. (2014). Safeguard power as a protection against imprecise power estimates. *Perspectives on Psychological Science*, *9*(3), 319–332. https://doi.org/10.1177/1745691614528519
